# Integrated proteomic and metabolomic profiling characterizes tryptophan metabolic disturbance associated with mortality in ICU-admitted SFTS patients

**DOI:** 10.3389/fimmu.2026.1898053

**Published:** 2026-10-09

**Authors:** Li Chen, Puhui Liu, Chuanzhen Niu, Chenxiao Hao, Fengxue Zhu, Guangjie Wang, Huiying Zhao

**Affiliations:** 1 Department of Critical Care Medicine, Peking University People’s Hospital, Beijing, China; 2 Department of Critical Care Medicine, Yantai Qishan Hospital, Yantai, Shandong, China

**Keywords:** metabolic dysregulation, metabolite-protein signatures, mortality, severe fever with thrombocytopenia syndrome, therapeutic targets

## Abstract

Severe fever with thrombocytopenia syndrome (SFTS), caused by Dabie bandavirus, is associated with high mortality among intensive care unit (ICU) patients; however, the integrated proteomic–metabolomic mechanisms underlying fatal outcomes remain poorly characterized. This prospective cohort study enrolled 68 patients with SFTS, who were admitted to the ICU and classified them as survivors (n = 33) or non-survivors (n = 35) according to their 28-day clinical outcomes. Untargeted metabolomic profiling using ultra-high-performance liquid chromatography–tandem mass spectrometry and quantitative proteomic profiling using data-independent acquisition mass spectrometry were performed on plasma samples collected at ICU admission. Multivariable analysis, pathway enrichment analysis, correlation analysis, and receiver operating characteristic (ROC) curve analysis were used to identify potential prognostic biomarkers and characterize molecular associations. Non-survivors exhibited more severe multi-organ injury and systemic inflammation. Metabolomic profiling revealed marked disruption of tryptophan metabolism: L-tryptophan levels were decreased, whereas kynurenine and its downstream metabolites (kynurenic acid [KA], 3-hydroxyanthranilic acid [3-HAA], xanthurenic acid [XA], and 5-hydroxyindolepyruvate [5-HIP]) were increased, suggesting activation of the kynurenine pathway. The four core tryptophan metabolites yielded a combined area under the curve (AUC) of 0.934 for predicting 28-day mortality and were correlated with indicators of renal function and coagulation. Proteomic profiling identified 200 differentially expressed proteins: proteins upregulated in survivors were enriched in the proteasome pathway, whereas those upregulated in non-survivors were enriched in the IL-17 pathway and other inflammatory and immune pathways. Integrated analysis revealed that the four tryptophan metabolites were associated with 20 differentially expressed proteins, six of which showed robust associations within outcome subgroups. Collectively, tryptophan metabolic disturbances coupled with inflammatory proteomic features may characterize fatal severe SFTS, and the identified metabolite–protein signatures may serve as promising prognostic biomarkers and potential therapeutic targets in critically ill patients.

## 1 Introduction

Severe fever with thrombocytopenia syndrome (SFTS) is a zoonotic disease caused by Dabie bandavirus, formerly known as SFTSV or the novel bunyavirus. It is predominantly transmitted by tick bites, particularly those of *Haemaphysalis longicornis*, and can also spread through direct contact with the blood or body fluids of infected patients, posing a risk of transmission and clustered outbreaks (1). First identified in China in 2009, SFTS has emerged as a major public health threat in East Asia, with endemic areas extending across China, Japan, South Korea, and Vietnam (2). Clinically, SFTS is characterized by acute high fever, marked thrombocytopenia and leukopenia, and multi-organ injury (3). Severe cases can progress rapidly to hemorrhagic diathesis, altered mental status, shock, and disseminated intravascular coagulation (4). Critically ill patients with concurrent multi-organ failure and central nervous system involvement have an extremely high mortality rate of up to 79.3%, whereas the mortality among ICU-admitted patients is approximately 44.7%, substantially exceeding the 10%–30% case-fatality rate reported in general SFTS cohorts (4). Currently, no specific antiviral agent or licensed vaccine is available, and clinical management remains largely supportive (5).

In viral infectious diseases, conventional clinical indicators and pathogen-detection methods are insufficient to comprehensively characterize the complex molecular interactions between viruses and their hosts (6). Proteomics can systematically characterize differential protein expression, post-translational modifications, and immune regulatory pathways during viral infection, thereby providing insights into host immune dysfunction and the molecular mechanisms underlying inflammatory injury (7). In contrast, metabolomics can dynamically characterize endogenous metabolic perturbations and pathway reprogramming associated with viral replication, providing biochemical insights into organ injury and disease severity (8). The integrated application of proteomics and metabolomics is a well-established and powerful multi-omics strategy for investigating the pathogenesis of emerging RNA viruses, identifying biomarkers of disease severity, and exploring potential therapeutic targets, and its utility has been demonstrated in various severe viral infections, including coronavirus and influenza virus infections (9, 10). However, the systematic molecular landscape of host metabolic and proteomic responses in patients with severe SFTS remains poorly understood. Key molecular markers associated with critical deterioration and multi-organ failure have not been fully identified and validated, thereby hindering the development of targeted treatment strategies for SFTS. Therefore, integrative proteomic and metabolomic analyses of patients with SFTS are warranted to address this research gap.

In this study, we comprehensively characterized the plasma proteomic and metabolomic profiles of patients with severe SFTS admitted to the intensive care unit (ICU), revealing coordinated proteomic and metabolic dysregulation during severe SFTS. These findings provide a foundation for further research into the diagnosis, treatment, and molecular mechanisms of this life-threatening disease.

## 2 Materials and methods

### 2.1 Study design and participants

This prospective cohort study included patients with severe SFTS who were admitted to the ICU between July 2019 and October 2023. According to their 28-day clinical outcomes, patients were divided into survivor (n = 33) and non-survivor (n = 35) groups. All specimens were collected in the Department of Critical Care Medicine, Yantai Qishan Hospital. The study was approved by the Ethics Review Committee of Yantai Qishan Hospital (approval no. 202503). The participants were informed about the research and its potential risks and provided written informed consent in accordance with the Declaration of Helsinki. The inclusion criteria (11) were as follows: (1) detection of novel bunyavirus nucleic acids in blood samples, confirmed by reverse transcription polymerase chain reaction (RT-PCR) in the laboratory, and (2) fulfillment of the criteria for severe and critical disease classification outlined in the *Consensus on Diagnosis and Treatment of Severe Fever with Thrombocytopenia Syndrome (2022 Edition).* The exclusion criteria were as follows: (1) clinically suspected severe SFTS with an alternative diagnosis established subsequently; (2) incomplete clinical data; (3) age <16 years; (4) immunocompromised status, defined as glucocorticoid or immunosuppressant use within 1 month, human immunodeficiency virus infection, solid or hematological malignancy, long-term nursing home residency, or hospitalization within 90 days; and (5) co-infection with severe acute respiratory syndrome coronavirus 2, hepatitis C virus, *Brucella, Mycobacterium tuberculosis*, or *Treponema pallidum* to avoid potential confounding.

### 2.2 Clinical data and plasma sample collection

Baseline demographic and clinical data were obtained from electronic medical records, including age, sex, comorbidities, heart rate, maximum body temperature, respiratory rate, and the interval between disease onset and ICU admission. The lowest Glasgow Coma Scale (GCS) score within 24 h before ICU admission and before sedation was recorded, along with altered mental status, bleeding events, and secondary infections. The primary endpoint was 28-day mortality after ICU admission. Laboratory parameters measured within 24 h of ICU admission included viral load, lactate, and other relevant laboratory indicators. Peripheral venous blood samples were collected in the morning within 24 h after ICU admission. All enrolled patients were fasting at the time of sampling. Blood was collected in anticoagulant tubes and immediately transported to the laboratory, where the samples were centrifuged at 1, 500 × g for 15 min. The upper plasma layer was collected and stored at −80 °C until further analysis. Before analysis, plasma samples were thawed on ice at 4 °C. To ensure sample quality, all samples included in this study underwent only one freeze–thaw cycle. To minimize potential bias, the sample-processing and LC-MS/MS injection orders were randomized between the survivor and non-survivor groups.

### 2.3 Metabolite extraction and LC-MS/MS analysis

All plasma samples were thawed on ice and vortexed vigorously. Hydrophilic metabolites were extracted using pre-cooled methanol. Briefly, 100 μL of homogenized plasma was mixed with 300 μL of pre-cooled methanol, vortexed thoroughly, and incubated at −20 °C for 30 min. The supernatants were then collected for instrumental analysis. Chromatographic separation was performed using a SHIMADZU-LC30 ultra-high-performance liquid chromatography system equipped with an ACQUITY UPLC^®^ HSS T3 column (2.1×100 mm, 1.8 μm) (Waters, Milford, MA, USA) and mass spectrometric analysis was performed using a QE Plus mass spectrometer (Thermo Fisher Scientific) with electrospray ionization in both positive and negative modes (**Supplementary Information File S1**). Raw mass spectrometry data were processed using MSDIAL software for peak alignment, retention-time correction, and peak-area extraction. Metabolite structures were identified by accurate-mass matching (mass tolerance <20 ppm) and MS2 spectrum matching (mass tolerance <0.01 Da) against public databases, including the Human Metabolome Database (HMDB), and a self-established in-house database. Ion peaks with >50% missing values within either group were excluded from subsequent statistical analyses. Before statistical analysis, all data were normalized to the total peak area to improve comparability and reduce variation.

To monitor analytical stability, quality control (QC) samples prepared by pooling equal volumes of all plasma samples were injected after every 15 experimental samples. QC assessment included comparison of base peak chromatograms of QC samples in both positive and negative ion modes, with consistent peak intensities and retention times across injections indicating minimal instrument variation, as well as principal component analysis of QC samples, which showed tight clustering and satisfactory repeatability. Because all samples were analyzed in a single batch and QC evaluation indicated stable instrument performance, no inter-batch correction was required.

### 2.4 Proteome sample preparation and data-independent acquisition mass spectrometry data analysis

Samples were thawed at 4 °C, and 2 μL of each sample was dissolved in 48 μL of UA buffer, incubated at 37 °C for 1 h, sonicated for 2 min, centrifuged, and then incubated at 4 °C for 20 min. The supernatants were collected. Low-abundance proteins were enriched using a low-abundance protein enrichment kit. An appropriate volume of 1 M DTT was added to the enriched samples to achieve a final concentration of 100 mM, followed by incubation at 37 °C for 30 min and cooling to 24 °C. Subsequently, an appropriate volume of IAA was added to achieve a final concentration of 50 mM, and the samples were incubated at room temperature for 30 min in the dark. Each sample was diluted sixfold with 50 mM NH_4_HCO_3_, after which 8 μg of Trypsin was added, and the resulting mixture was vortexed at 600 rpm for 1 min and incubated at 37 °C for 20 h. The enzymatically digested samples were acidified with an appropriate volume of TFA, and the peptides were desalted using a C18 cartridge and reconstituted in 0.1% FA; peptide concentrations were then determined before LC-MS analysis. Appropriate amounts of peptides from each sample were separated using a Vanquish Neo UHPLC system (Thermo Fisher Scientific) (**Supplementary Information File S2**).

After peptide separation, DIA mass spectrometry was performed using an Orbitrap Astral mass spectrometer (Thermo Fisher Scientific). The total analysis duration was 8 min, with an electrospray voltage of 2.2 kV in positive-ion detection mode. The precursor-ion scan range was 380–980 m/z, the full scan resolution was 240, 000, the AGC target was 500%, and the maximum ion injection time (IT) for the full scan was 3 ms. The MS2 parameters were as follows: resolution, 80, 000; AGC target, 500%; maximum IT, 3 ms; RF lens, 40%; activation type, HCD; isolation window, 2 Th; normalized collision energy, 25%; and cycle time, 0.6 s.

All mass spectrometry data were processed using DIA-NN software for database searching and quantitative analysis of the DIA mass spectrometry data. The database used was the Swiss-Prot-Homo sapiens (Human) [9606]-reviewed-20421-20250908-iRT.fasta database. Subsequently, statistical analyses were performed on the qualitative and quantitative DIA proteomic results. Each sample was prepared independently and, after enzymatic protein digestion, subjected to DIA analysis. The resulting raw DIA mass spectrometry files were imported into DIA-NN for analysis using a q value threshold of ≤0.01.

### 2.5 Omics data processing, bioinformatics, and statistical analysis

Proteins and metabolites with valid measurements in fewer than 50% of samples in either group were excluded from further analyses. For metabolomics, differential metabolites were identified using the OPLS-DA model with variable importance in projection (VIP) >1.0, combined with univariate analysis (*P* < 0.05), an approach suited to the high-dimensional and multicollinear nature of metabolomic data. Metabolites were compared between survivors and non-survivors. Based on the intensity ratio of survivors/non-survivors, up-regulated metabolites denote higher abundance in non-survivors, while down-regulated metabolites denote higher abundance in survivors. For proteomics, differentially expressed proteins were defined based on a fold-change threshold (|FC| ≥ 1.5) and *P* < 0.05. The FC-based criterion was adopted because proteomic datasets typically contain fewer and more directly interpretable features than metabolomic datasets, for which multivariable filtering (e.g., VIP) is more commonly applied.

Correlation analysis was used to evaluate associations between the four tryptophan metabolites and plasma proteins, as well as between the metabolites and clinical parameters. To determine whether these associations were independent of intergroup differences, correlations were calculated separately in the overall cohort (n = 68) and in the survivor (n = 33) and non-survivor (n = 35) groups. Within each stratum, *P* values were adjusted for multiple testing using the Benjamini–Hochberg method to control the FDR. In the overall cohort, proteins with |r| ≥0.35 and *P* < 0.05 were considered significantly correlated with the corresponding metabolite and were eligible for inclusion in the integrated metabolite–protein network. Associations that remained significant (*P* < 0.05) within either subgroup were defined as robust associations.

Receiver operating characteristic (ROC) curves were constructed to assess the discriminative value of differential metabolites and proteins for 28-day mortality in patients with severe SFTS. The area under the curve (AUC), sensitivity, specificity, and 95% confidence interval (95% CI) were calculated. Categorical variables are presented as frequencies and percentages, whereas continuous variables are expressed as medians with interquartile ranges (IQRs). Continuous variables were compared between groups using the Wilcoxon rank-sum test or Kruskal–Wallis test, whereas categorical variables were analyzed using the chi-square test or Fisher’s exact test. Multiple-testing correction was performed using the Benjamini–Hochberg method. All statistical tests were two-tailed, and *P* < 0.05 was considered statistically significant. All analyses were performed using R, SPSS version 19.0, MedCalc version 15.8, and MetaboAnalyst version 4.0 (Wishart Research Group, University of Alberta, Canada). (http://www.metaboanalyst.ca/).

## 3 Results

### 3.1 Participant demographics and clinical data

The patient enrollment and exclusion process is summarized in the flow diagram (**Supplementary Information Figure S1**). A total of 68 patients were enrolled in this study, and the baseline characteristics were compared between the 28-day survivor (n = 33) and non-survivor (n = 35) groups. No statistically significant difference were observed between the two groups in demographic characteristics (e.g., age and sex) or most comorbidities (e.g., diabetes mellitus, hypertension, and coronary heart disease) (all *P* > 0.05).

However, regarding clinical and laboratory parameters, patients in the non-survivor group exhibited a more severe disease phenotype, characterized by a significantly lower median GCS (9.0 vs. 11.0, *P* = 0.002) and a substantially higher median viral RNA copy number (1, 150, 000 vs. 8, 138, *P* < 0.001). Moreover, the non-survivor group showed a more pronounced systemic inflammatory response, as evidenced by significantly elevated levels of lactate, PCT, IL-6, and ferritin (all *P* < 0.05). Regarding organ dysfunction, this group had significantly higher levels of liver function markers (ALT, AST, and ALP), renal function parameters (BUN, Cr, and Cys-C), and myocardial injury indicators (hsTnI and BNP) (all *P* < 0.05). Regarding coagulation function, the non-survivor group had significantly prolonged PT, APTT, INR, and TT, along with a markedly lower FIB level and significantly elevated D-dimer levels (all *P* < 0.05). Detailed baseline demographic characteristics and clinical information for all participants are presented in **Table 1**.

**Table 1:** Baseline demographic and clinical characteristics of the study population.

Characteristic	Survivors (n = 33)	Non-survivors (n = 35)	*P* value
Demographics and vital signs			
Age, y	72.00 (62.00-77.00)	72.00 (67.00-76.00)	0.834
Female, n (%)	17 (51.52)	15 (42.86)	0.475
Body mass index, kg/m²	24.91 (23.66-25.71)	24 (22.87-25.52)	0.121
Heart rate, beats/min	87 (65-98)	83 (68-102)	0.672
Respiratory rate, breaths/min	20 (20-25)	25 (19-30)	0.133
Maximum temperature, °C	39.00 (38.50-39.30)	38.70 (38.30-39.00)	0.098
Comorbidities and lifestyle factors			
Any underlying disease, n (%)	26 (78.79)	22 (62.86)	0.150
Hypertension, n (%)	14 (42.42)	9 (25.71)	0.145
Diabetes mellitus, n (%)	8 (24.24)	10 (28.57)	0.686
Coronary heart disease, n (%)	4 (12.12)	5 (14.29)	0.999
Smoking, n (%)	5 (15.15)	8 (22.86)	0.419
Alcohol consumption, n (%)	6 (18.18)	11 (31.42)	0.207
Clinical manifestations			
Altered consciousness, n (%)	25 (75.76)	31 (88.57)	0.166
Jaw or limb tremor, n (%)	24 (72.73)	22 (62.86)	0.385
Myalgia, n (%)	18 (64.55)	17 (48.57)	0.622
Anorexia, n (%)	33 (100)	32 (91.43)	0.239
Lymphadenopathy, n (%)	6 (18.18)	4 (11.43)	0.507
Bleeding, n(%)	10 (30.30)	22 (62.86)	0.007
Gastrointestinal symptoms, n (%)	22 (66.67)	22(62.86)	0.743
cardiac_symptoms, n (%)	20 (60.61)	27 (77.14)	0.140
Glasgow Coma Scale score	11.00 (9.00-13.00)	9.00 (7.00-10.00)	0.002
Laboratory findings at admission			
WBC, ×10^9^/L	3.40 (2.20-8.70)	3.80 (2.40-10.00)	0.663
RBC, ×10¹²/L	4.46 (4.04-4.81)	4.63 (4.18-5.09)	0.384
Hb, g/L	134.00 (126.00-142.00)	138.00 (123.00-155.00)	0.308
Plt, ×10^9^/L	48.00 (35.00-64.00)	45.00 (32.00-58.00)	0.418
NE%, %	61.00 (50.00-77.00)	68.00 (55.00-82.00)	0.232
NE#, ×10^9^/L	2.54 (1.26-5.79)	2.89 (1.69-5.34)	0.500
LY#, ×10^9^/L	0.73 (0.49-1.59)	0.62 (0.41-1.08)	0.458
MO#, ×10^9^/L	0.46 (0.14-1.29)	0.38 (0.12-1.16)	0.650
EO, %	0.10 (0.00-0.10)	0.00 (0.00-0.10)	0.619
ALT, U/L	80.00 (43.00-143.00)	157.00 (69.00-281.00)	0.003
AST, U/L	178.00 (94.00-314.00)	513.00 (185.00-906.00)	<0.001
GGT, U/L	37.00 (23.00-170.00)	73.00 (36.00-204.00)	0.056
ALP, U/L	56.00 (44.00-80.00)	91.00 (63.00-156.00)	<0.001
Dbil, μmol/L	3.00 (2.00-6.00)	7.00 (3.00-14.00)	0.017
IBil, μmol/L	6.58 (5.15-8.45)	7.04 (5.61-8.22)	0.955
TBil, μmol/L	9.00 (7.00-13.00)	16.00 (10.00-23.00)	0.097
Total protein, g/L	56.60 (53.40-59.80)	56.60 (53.40-59.80)	0.411
TC, mmol/L	3.30 (2.77-3.70)	3.00 (2.53-4.01)	0.646
TG, mmol/L	1.99 (1.13-3.09)	1.63 (1.00-1.96)	0.191
HDL-C, mmol/L	0.80 (0.60-0.87)	0.80 (0.64-0.94)	0.568
LDL-C, mmol/L	1.65 (0.94-1.88)	1.37 (1.09-1.99)	0.869
GLU, mmol/L	8.40 (6.40-13.70)	9.00 (6.40-12.40)	0.768
Cr, μmol/L	60.00 (48.00-77.00)	104.00 (67.00-195.00)	<0.001
BUN, mmol/L	6.70 (5.20-9.30)	12.10 (7.00-17.60)	<0.001
UA, μmol/L	293.00 (213.00-364.00)	399.00 (299.00-457.00)	0.004
Cystatin C, mg/L	1.10 (1.02-1.65)	2.37 (1.56, 3.74)	<0.001
AMY, U/L	82.00 (65.00-134.00)	108.00 (78.00-228.00)	0.058
CHE, U/L	5, 131 (4, 331, 6, 494)	4, 597 (4, 072, 5, 175)	0.130
Homocysteine, μmol/L	9.50 (7.50-13.70)	12.70 (11.10-14.60)	0.012
APTT, s	49 (45-52)	69 (60-87)	<0.001
TT, s	26 (22-30)	41 (27-73)	<0.001
PT, s	13.20 (12.90-13.60)	14.00 (13.20-15.60)	<0.001
FIB, g/L	2.60 (2.30-3.22)	1.99 (1.72-2.29)	<0.001
INR	0.99 (0.96-1.03)	1.06 (1.00-1.21)	<0.001
DD, mg/L(FEU)	2 (1-3)	4 (2-11)	0.011
PH	7.41 (7.37-7.45)	7.37 (7.28-7.41)	0.012
PaO2, mmHg	104 (81-118)	89 (74-114)	0.155
PaCO2, mmHg	34 (30-39)	33 (27-37)	0.581
HCO3-, mmol/L	22.10 (20.20-24.0)	18.70 (16.20-21.90)	<0.001
PaO_2_/FiO_2_ ratio, mmHg	257 (175-333)	192 (142-257)	0.038
K, mmol/L	3.93 (3.63-4.18)	4.30 (3.90-4.80)	0.002
Ca, mmol/L	1.92 (1.78-2.00)	1.85 (1.73-2.01)	0.888
Lactate, mmol/L	1.00 (0.80-1.50)	2.10 (1.60-3.80)	<0.001
Procalcitonin, ng/mL	0.20 (0.10-0.60)	0.90 (0.30-3.30)	0.003
High-sensitivity CRP, mg/L	8 (3-17)	15 (4-42)	0.143
Serum amyloid A, mg/L	59 (42-206)	155 (78-303)	0.039
Sialic acid, mg/L	552 (507- 600)	527 (472- 652)	0.969
CD4+, cells/µL	179 (83-299)	77 (47-147)	0.002
CD8+, cells/µL	209 (80-501)	104 (75-171)	0.042
hsTnI, ng/L	126 (39-385)	213 (66-1, 526)	0.048
CK-MB, U/L	4 (2-7)	6 (4-11)	0.170
BNP, pg/mL	167 (66-267)	358 (154-668)	0.015
maximum viral RNA copies	8, 138 (1, 270-89, 200)	1, 150, 000 (15, 000-11, 300, 000)	<0.001

WBC, white blood cell count; RBC, red blood cell count; Hb, hemoglobin; Plt, platelet count; NE%, neutrophil percentage; NE#, absolute neutrophil count; LY#, absolute lymphocyte count; MO#, absolute monocyte count; EO%, eosinophil percentage; ALT, alanine aminotransferase; AST, aspartate aminotransferase; GGT, γ-glutamyl transferase; ALP, alkaline phosphatase; Dbil, direct bilirubin; IBil, indirect bilirubin; TBil, total bilirubin; Total protein, total serum protein; TC, total cholesterol; TG, triglycerides; HDL-C, high-density lipoprotein cholesterol; LDL-C, low-density lipoprotein cholesterol; GLU, blood glucose; Cr, creatinine; BUN, blood urea nitrogen; UA, uric acid; Cystatin C, cystatin C; AMY, amylase; CHE, cholinesterase; Homocysteine, homocysteine; APTT, activated partial thromboplastin time; TT, thrombin time; PT, prothrombin time; FIB, fibrinogen; INR, international normalized ratio; DD, D-dimer; pH, potential of hydrogen; PaO_2_, arterial partial pressure of oxygen; PaCO_2_, arterial partial pressure of carbon dioxide; HCO_3_^−^, bicarbonate; K, potassium; Ca, calcium; Lactate, blood lactate; Procalcitonin, procalcitonin; High-sensitivity CRP, high-sensitivity C-reactive protein; Serum amyloid A, serum amyloid A protein; Sialic acid, sialic acid; CD4^+^, CD4^+^ T lymphocyte count; CD8^+^, CD8^+^ T lymphocyte count; LDH, lactate dehydrogenase; hsTnI, high-sensitivity troponin I; CK, creatine kinase; CK-MB, creatine kinase-MB; BNP, B-type natriuretic peptide.

### 3.2 Metabolic landscape of fatal outcomes characterized by severe dysregulation and disrupted tryptophan metabolism

A total of 21, 761 ion peaks were detected in both positive and negative ion modes. Principal component analysis (PCA), an unsupervised multivariable analysis, revealed tight clustering of the quality control samples, indicating good analytical reproducibility and instrument stability (**Figure 1a**). Orthogonal partial least squares discriminant analysis (OPLS-DA) demonstrated clear separation between the groups, suggesting good discriminatory performance (**Figure 1b**). Permutation testing further confirmed the robustness of the model, with Q² > 0.8 and R²Y > 0.8 (**Supplementary Information Figure S2**). Overall, 819 differential metabolites were identified at levels 1 and 2 based on a variable importance in projection (VIP) >1 and *P* < 0.05 (**Figure 1c**). Compared with the survivor group, 296 metabolites were upregulated and 523 were downregulated in the non-survivor group (**Figure 1d**). Hierarchical clustering of the top 50 metabolites ranked by VIP score revealed distinct metabolic profiles (**Figure 1e**).

**Figure 1:**
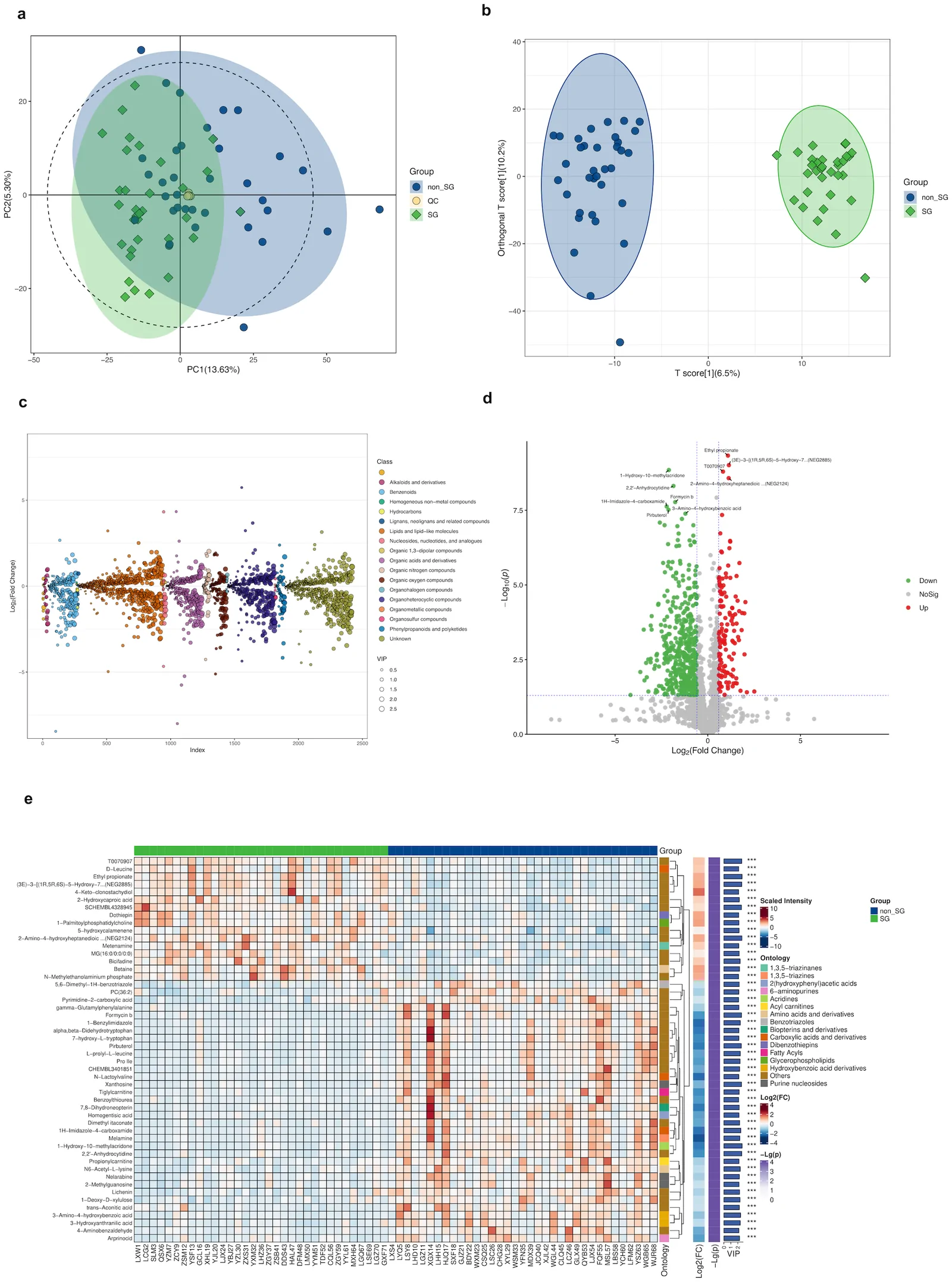
Plasma metabolomic profiling reveals distinct metabolic profiles between survivors and non-survivors. **(A)** PCA score plot: PC1 (13.63%) and PC2 (5.30%). Quality control (QC) samples clustered tightly near the origin, indicating high reproducibility and instrument stability; non-SG (non-survivors) and SG (survivors) were separated along PC1. **(B)** OPLS-DA score plot: T score [1] (6.5%) versus Orthogonal T score [1] (10.2%); non-SG and SG were clearly separated, demonstrating robust discriminative capability. **(C)** Distribution of differential metabolites: x-axis, metabolite index; y-axis, Log2(fold change); dot color represents chemical class (16 classes) and dot size represents the VIP value (0.5–2.5). **(D)** Volcano plot: Log2(FC) versus −Log10(P); red, up-regulated; green, down-regulated; gray, not significant; representative metabolites are labeled. **(E)** Hierarchical clustering heatmap of the top 50 VIP metabolites. Each row represents one metabolite (name shown on the left), and each column represents one sample, with the top color bar indicating group (green, SG; dark blue, non-SG). Rows and columns were clustered using the complete method with Euclidean distance; the row dendrogram groups metabolites with similar expression patterns, and the column dendrogram assesses whether samples separate naturally by group. Colors represent scaled metabolite signal intensity (red/orange, high abundance; blue, low abundance). The four right-side annotation bars show: (i) Ontology, chemical classification of metabolites (e.g., amino acids, fatty acids, glycerophospholipids, carboxylic acid derivatives); (ii) Log2(FC), log2-transformed fold change (red, up-regulated; blue, down-regulated) reflecting the magnitude of between-group change; (iii) −Lg(p), negative logarithm of the P value—taller bars indicate greater statistical significance (***significant between-group difference); and (iv) VIP, variable importance in projection from the OPLS-DA model—higher values indicate greater contribution to group discrimination and potential biomarker value (***significant).

Multiple metabolic pathways associated with fatal outcomes (**Supplementary Information Table S1**). Among the level-1 pathway categories, metabolism, cellular processes, and other related biological processes were perturbed. Within these categories, tryptophan, tyrosine, and pyrimidine metabolism were significantly dysregulated in non-survivors, with tryptophan metabolism showing the most pronounced alterations (**Figure 2**). Specifically, L-tryptophan was significantly decreased in non-survivors, whereas key intermediates of the kynurenine pathway, including kynurenine, kynurenic acid (KA), xanthurenic acid (XA), 3-hydroxyanthranilic acid (3-HAA), and 5-hydroxyindolepyruvate (5-HIP), were significantly increased (**Figure 2**). These results yield a metabolite pattern consistent with enhanced kynurenine pathway activity in fatal SFTS.

**Figure 2:**
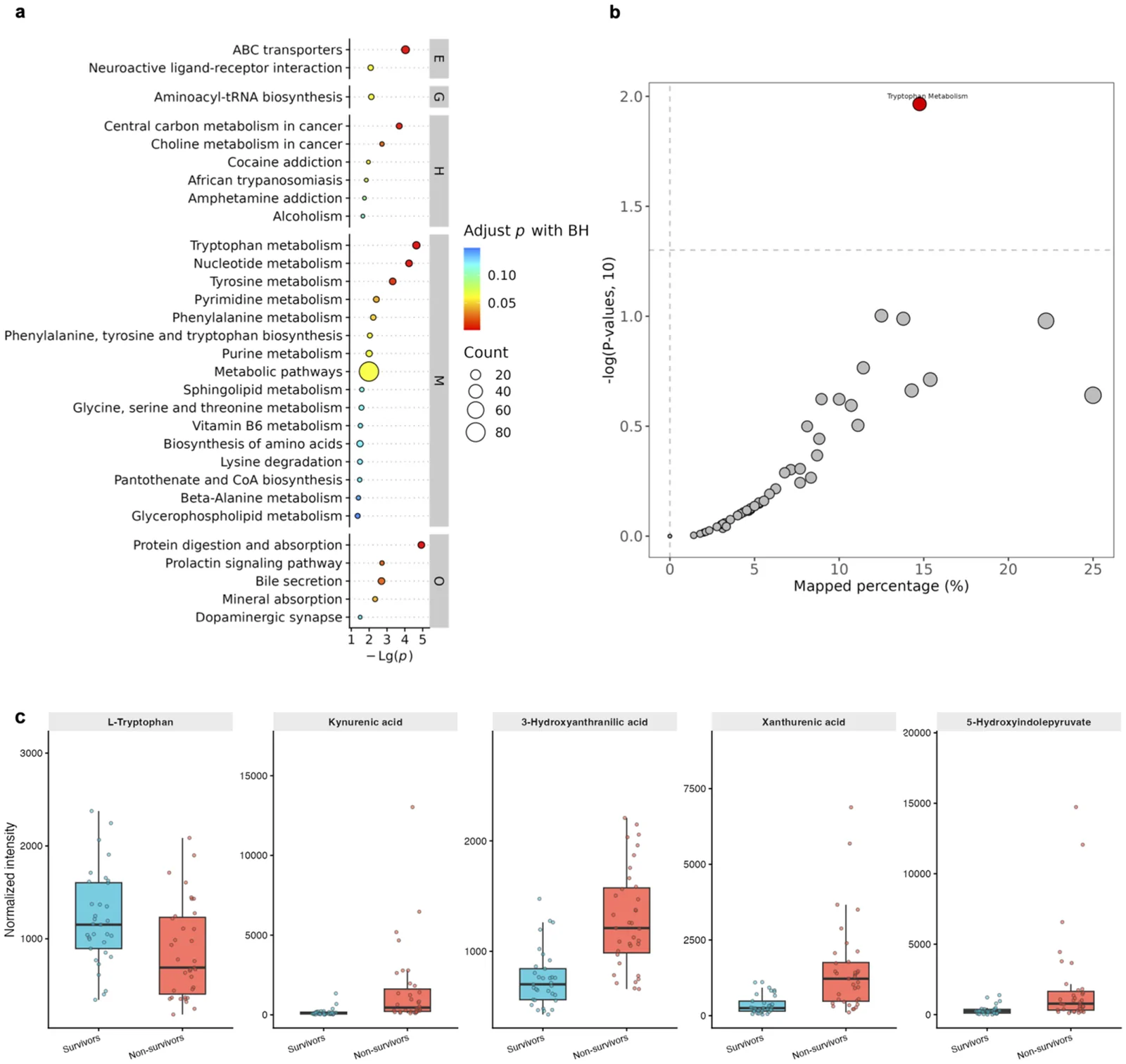
Pathway enrichment of differential metabolites and comparison of core tryptophan metabolites between groups. **(A)** KEGG pathway enrichment bubble plot of differential metabolites: x-axis, −Lg(p); bubble size, number of enriched metabolites (Count); color, BH-adjusted P value. Tryptophan metabolism (10/106 metabolites; adjusted P = 0.001) was the most significantly enriched pathway. **(B)** Enrichment scatter plot: x-axis, Mapped percentage (%); y-axis, −log(P-values, 10); tryptophan metabolism (red) was prominently enriched. **(C)** Boxplots of normalized intensities of L-tryptophan and the four core tryptophan metabolites (kynurenic acid [KA], 3-hydroxyanthranilic acid [3-HAA], xanthurenic acid [XA], and 5-hydroxyindolepyruvate [5-HIP]) in survivors (blue) and non-survivors (red); FC, P, FDR, and VIP values are indicated above each panel (L-Trp: FC(non-SG/SG) = 0.71, P = 0.0067, FDR = 0.031, VIP = 1.23; KA: FC(non-SG/SG) = 9.09, P = 0.0031, FDR = 0.0175, VIP = 1.40; 3-HAA: FC(non-SG/SG) = 1.72, P < 0.001, FDR < 0.001, VIP = 2.36; XA: FC(non-SG/SG) = 4.00, P < 0.001, FDR < 0.001, VIP = 1.86; 5-HIP: FC(non-SG/SG) = 6.25, P = 0.0054, FDR = 0.0268, VIP = 1.32).

### 3.3 Prognostic performance of tryptophan metabolites and clinical indicators

Among the 819 differential metabolites initially identified using OPLS-DA model (VIP score >1.0) combined with univariate analysis (*P* < 0.05), we prioritized four tryptophan-related metabolites for further prognostic evaluation based on their statistical significance and biological relevance. These four metabolites represent core metabolites of the tryptophan catabolic pathway.

To evaluate the prognostic value of these metabolites, we performed ROC curve analyses for all individual variables (**Supplementary Information Table S2**). Among the four metabolites, KA showed the highest discriminative performance for short-term mortality (AUC = 0.892, 95% CI: 0.817–0.966), followed by 3-HAA (AUC = 0.860, 95% CI: 0.774–0.946), XA (AUC = 0.850, 95% CI: 0.761–0.939), and 5-HIP (AUC = 0.810, 95% CI: 0.708–0.911). Notably, all four metabolites achieved AUC values > 0.80, whereas the conventional clinical indicators BNP (AUC = 0.675) and hsTnI (AUC < 0.60) showed lower AUC values. We next constructed a combined prognostic signature using the four metabolites through multivariable logistic regression. The four-metabolite panel achieved an AUC of 0.934 (95% CI: 0.878–0.991), indicating excellent discriminative ability (**Figure 3**). Among the clinical indicators, APTT showed the highest individual discriminative performance (AUC = 0.863, 95% CI: 0.772–0.954). Although the four-metabolite panel showed a numerically higher AUC than APTT, the difference was not statistically significant (DeLong *P* = 0.167), possibly because of the limited sample size (**Supplementary Information Table S2**).

**Figure 3:**
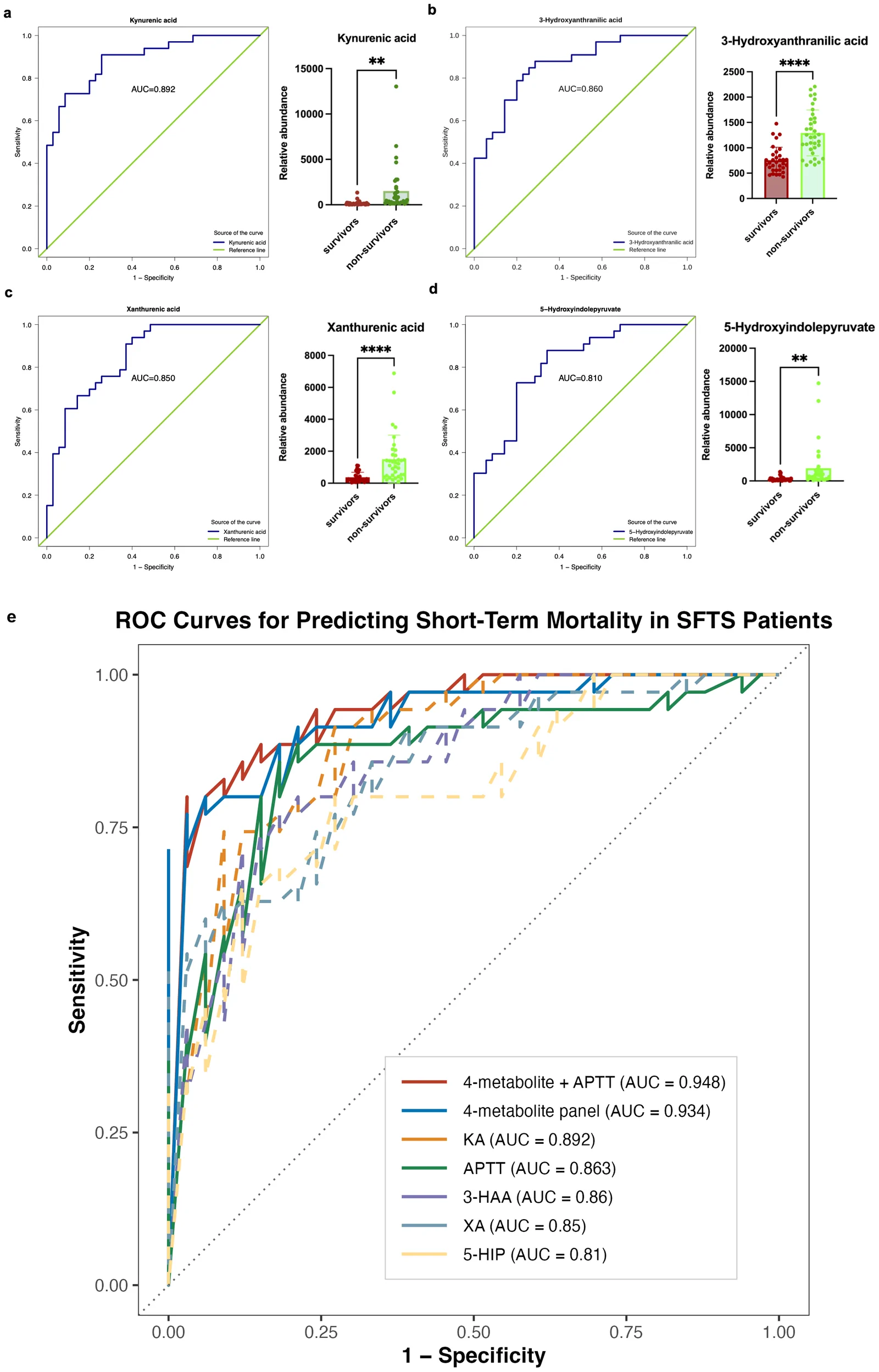
ROC analysis of tryptophan metabolites for predicting 28-day mortality. **(A–D)** ROC curves (left) and relative abundance boxplots (right, survivors versus non-survivors, with significance indicated) for kynurenic acid (AUC = 0.892), 3-hydroxyanthranilic acid (AUC = 0.860), xanthurenic acid (AUC = 0.850), and 5-hydroxyindolepyruvate (AUC = 0.810) in predicting 28-day mortality. **(E)** Combined ROC curves (ROC Curves for Predicting Short-Term Mortality in SFTS Patients): 4-metabolite + APTT (AUC = 0.948), 4-metabolite panel (AUC = 0.934), KA (0.892), APTT (0.863), 3-HAA (0.86), XA (0.85), and 5-HIP (0.81); gray dashed line, reference line.

To further explore whether integrating clinical information could improve discriminative performance, we combined the four-metabolite panel with APTT (**Figure 3**). The combined model yielded an AUC of 0.948 (95% CI: 0.902–0.994), which was significantly higher than that of APTT alone (ΔAUC = 0.085, DeLong *P* = 0.047). However, the addition of APTT did not significantly improve the performance of the four-metabolite panel alone (ΔAUC = 0.014, DeLong *P* = 0.360), suggesting that the addition of APTT provided limited incremental discriminative value for short-term mortality in this cohort.

### 3.4 Association of tryptophan metabolites with renal and coagulation dysfunction

To further characterize the clinical relevance of the identified tryptophan metabolites, we performed Pearson correlation analyses between the four metabolites and a comprehensive panel of clinical indicators in the overall cohort and after stratification by survival status (full results in **Supplementary Information Table S3**). Here, we highlight the correlations with BUN and APTT, two established indicators of disease severity in SFTS (**Figure 4**). In the overall cohort, all four metabolites were significantly positively correlated with APTT (r = 0.52–0.73, all *P* < 0.001), and three metabolites (KA, XA, and 5-HIP) were also significantly positively correlated with BUN (r = 0.53–0.57, all *P* < 0.001). Notably, stratified analysis indicated that these correlations were stronger in the non-survivor group. Among non-survivors, seven of the eight metabolite–indicator pairs were statistically significant, with XA showing the strongest correlation with BUN (r = 0.68, *P* < 0.001) and KA showing the strongest correlation with APTT (r = 0.59, *P* < 0.001). In contrast, among survivors, only two pairs (KA–APTT and 5-HIP–APTT) reached nominal significance, with weaker correlation coefficients (r = 0.35–0.54) (**Supplementary Information Table S3**). This group-dependent pattern suggests that the associations between tryptophan metabolites and clinical severity markers may reflect systematic differences between patients with distinct clinical outcomes rather than being attributable solely to a few outlier values.

**Figure 4:**
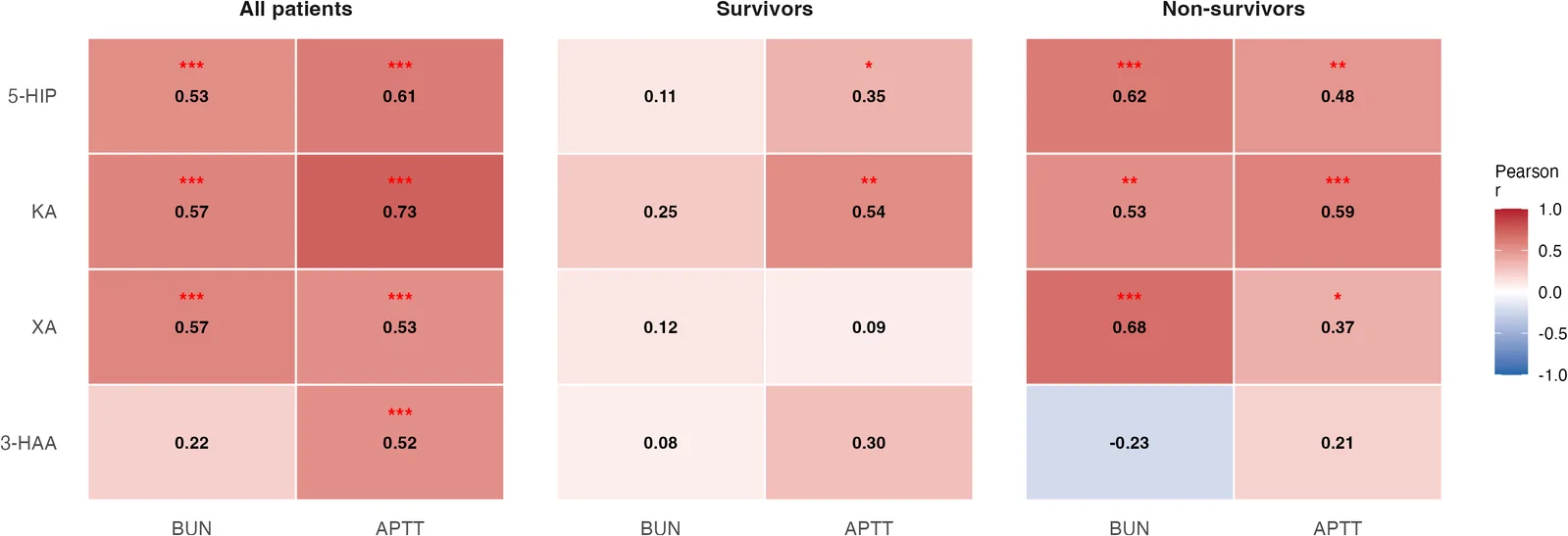
Correlation heatmap between tryptophan metabolites and clinical indicators. Pearson correlation coefficients between the four tryptophan metabolites (5-HIP, KA, XA, and 3-HAA) and clinical indicators (BUN and APTT), calculated in the overall cohort (All patients, n = 68), survivors (n = 33), and non-survivors (n = 35). Red, positive correlation; blue, negative correlation; r values and significance levels are shown within cells (*P < 0.05, **P < 0.01, ***P < 0.001).

Consistent with these findings, all four tryptophan metabolites were significantly higher in non-survivors than in survivors (**Figure 2c**; 3-HAA: *P* = 5.6 × 10^−8^; XA: *P* = 1.4 × 10^−7^; KA: *P* = 1.7 × 10^−9^; 5-HIP: *P* = 4.9 × 10^−6^), further supporting their association with disease severity and fatal outcomes in SFTS.

### 3.5 Plasma proteome dysregulation in fatal SFTS

Further proteomic profiling was conducted on plasma samples from the survivor (n = 33) and non-survivor (n = 35) groups, resulting in the identification of 6, 043 proteins. Quality control demonstrated that high mass accuracy was consistently maintained during data acquisition, supporting the reliability and stability of the proteomic data (**Figure 5a**). Comparative analysis between the two outcome groups identified 200 differentially expressed proteins (DEPs) (*P* < 0.05; fold change ≥1.5 or ≤0.67). Among these, 105 proteins showed significantly higher abundance in survivors, whereas 95 showed significantly higher abundance in non-survivors (**Figure 5b**).

**Figure 5:**
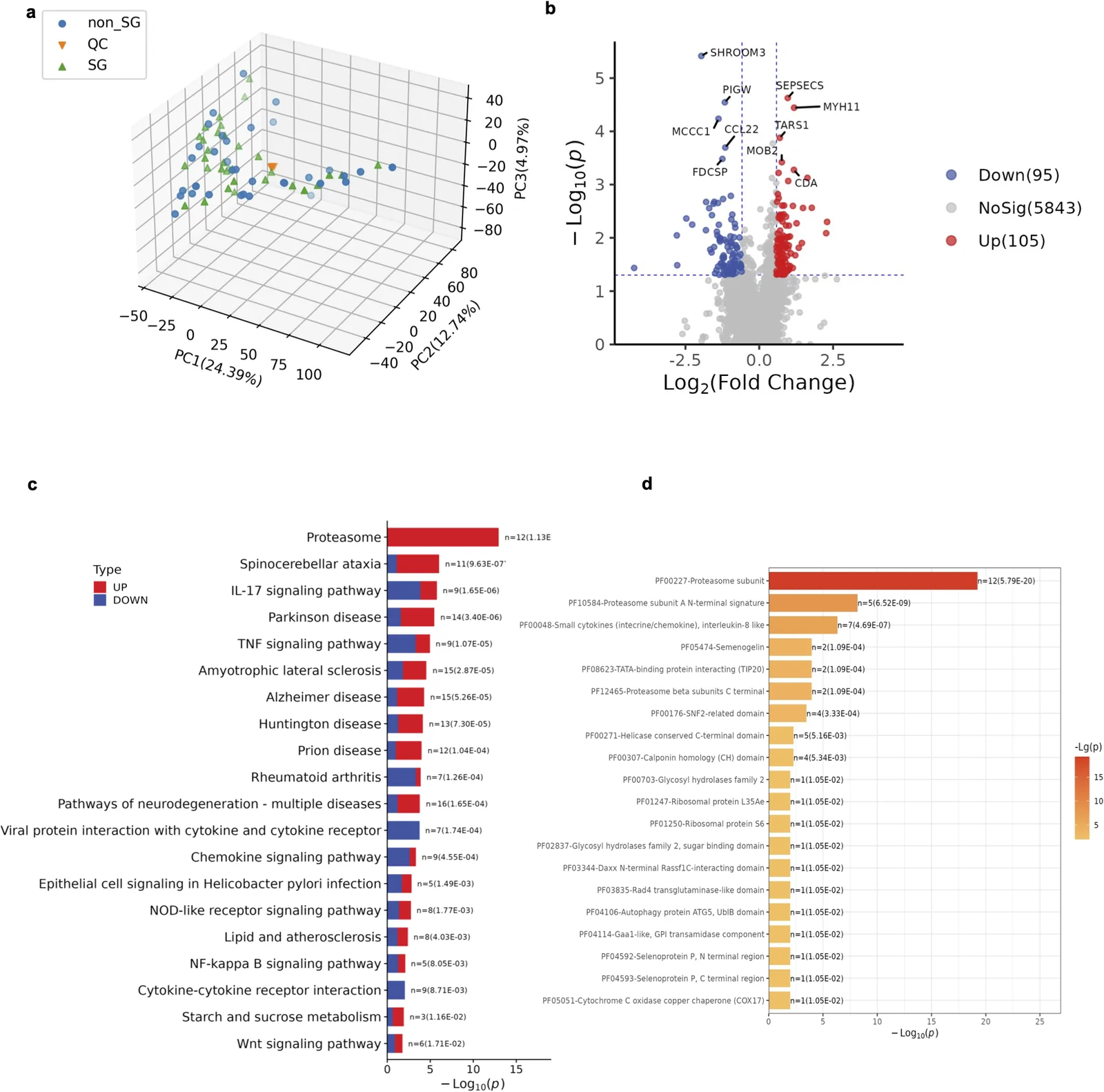
Plasma proteomic differential analysis. **(A)** 3D-PCA score plot of proteomic quality control: PC1, 24.39%; PC2, 12.74%; PC3, 4.97%. QC samples clustered tightly, confirming stable data quality. **(B)** Volcano plot of differentially expressed proteins (DEPs): Log2(FC) versus −Log10(P); red, up-regulated (Up, n = 105); blue, down-regulated (Down, n = 95); gray, not significant (NoSig, n = 5843); representative proteins are labeled (SHROOM3, PIGW, SEPSECS, MYH11, TARS1, MCCC1, CCL22, MOB2, FDCSP, and CDA). **(C)** KEGG pathway enrichment analysis of DEPs: x-axis, −Log10(P); red (UP) and blue (DOWN) represent pathways enriched by proteins up-regulated in each direction, with gene counts **(n)** and P values indicated. Major enriched pathways included the proteasome (n = 12), IL-17 signaling (n = 9), TNF signaling (n = 9), chemokine signaling (n = 9), and cytokine–cytokine receptor interaction (n = 9). **(D)** Pfam domain enrichment analysis of DEPs: x-axis, −Log10(P); the most significantly enriched domains were the proteasome subunit PF00227 (n = 12, P = 5.79E-20) and PF10584 (n = 5, P = 6.52E-09), followed by small cytokines PF00048 (n = 7, P = 4.69E-07).

KEGG pathway enrichment analysis revealed distinct enrichment patterns between the two groups of DEPs. Proteins upregulated in survivors were primarily enriched in the proteasome pathway and neurodegenerative disease-related pathways, with the proteasome pathway showing the strongest enrichment (**Figure 5c**). Conversely, proteins upregulated in non-survivors were predominantly associated with IL-17 signaling, TNF/NF-κB signaling, chemokine signaling, and cytokine–cytokine receptor interaction pathways, all of which are involved in inflammation and immune regulation (**Figure 5c**). These results suggest a heightened inflammatory response in patients with fatal outcomes, whereas protein homeostasis-related pathways were relatively preserved in survivors. Consistent with this, domain enrichment analysis revealed that proteasome subunits were the most significantly enriched domains among the proteins upregulated in survivors (**Figure 5d**), followed by domains associated with signal regulation, cytoskeletal organization, and vesicle transport.

To evaluate whether these molecular features could consistently distinguish survivors from non-survivors at the individual-sample level rather than being driven by a small subset of samples, we performed sample-level hierarchical clustering of the four tryptophan metabolites and the 200 DEPs. Features were z-score normalized row-wise, and columns were annotated by clinical outcome (**Supplementary Information Figure S3**; the complete list of DEPs is provided in **Supplementary Information Table S4**). Hierarchical clustering showed that survivors and non-survivors largely formed two relatively distinct clusters, with expression patterns showing outcome-consistent directional changes. The 105 proteins upregulated in survivors and the 95 proteins upregulated in non-survivors exhibited opposite expression gradients between the two groups, while the four tryptophan metabolites (KA, 3-HAA, XA, and 5-HIP) likewise showed outcome-consistent distribution patterns (**Supplementary Information Figure S3**). These findings suggest that the integrated metabolomic–proteomic features identified in this study may distinguish the two outcome groups and are not obviously attributable to individual extreme samples, further supporting the reliability of the intergroup differential analyses described above.

### 3.6 Integrated network of core tryptophan pathway metabolites and differentially expressed proteins

KEGG enrichment analysis of differential metabolites identified tryptophan metabolism as a key perturbed pathway (hsa00380; 10/106 metabolites, adjusted *P* = 0.001). The four core metabolites selected for outcome discrimination were consistently higher in non-survivors. To construct an integrated metabolite–protein network, Spearman correlation analysis was performed between the four core metabolites and the 200 DEPs in the overall cohort (n = 68). Twenty DEPs exhibited significant correlations with at least one core metabolite (|r| ≥ 0.35, *P* < 0.05), including MMP3, B4GALT5, SHROOM3, SEPSECS, and MYH11 (**Figure 6**; **Supplementary Information Table S5**). Because pooling all patients can inflate correlations that are driven primarily by between-group differences, correlations were recalculated separately in survivors (n = 33) and non-survivors (n = 35). Most correlations were attenuated within the subgroups, suggesting that they were influenced substantially by between-group differences. Notably, six metabolite–protein pairs remained significant after subgroup stratification: MMP3, SH3BGR, and BIN1 were negatively correlated with 3-HAA in survivors (r = −0.37 to −0.40, all *P* < 0.05), whereas CHL1–3-HAA (r = −0.34, *P* = 0.046), PLSCR3–XA (r = −0.38, *P* = 0.026), and MYOF–XA (r = −0.36, *P* = 0.032) remained significant in non-survivors (**Figure 6**).

**Figure 6:**
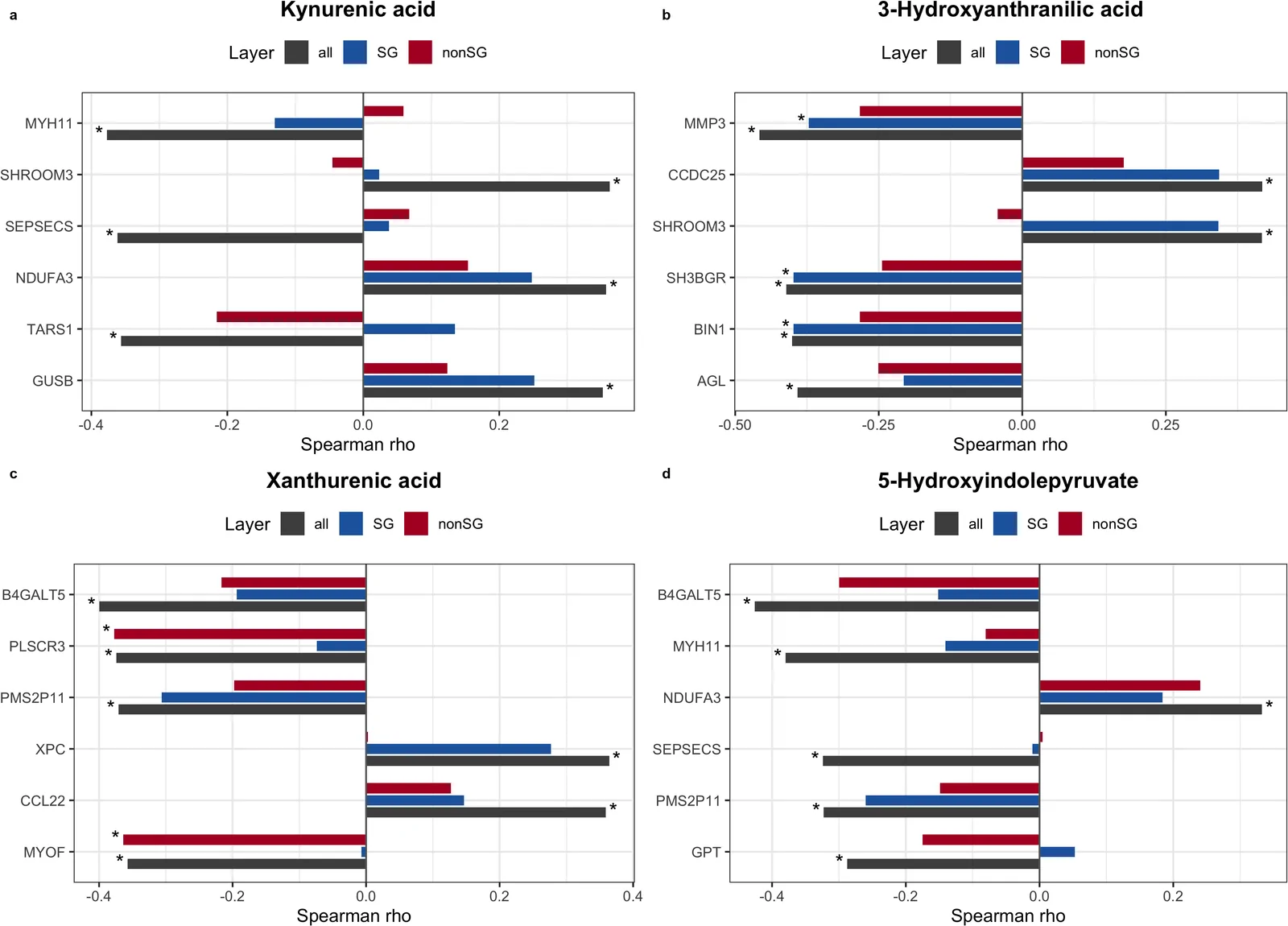
Spearman correlation analysis between tryptophan metabolites and differentially expressed proteins. **(A–D)** Bar charts of Spearman correlation coefficients for the proteins most strongly correlated with kynurenic acid **(A)**, 3-hydroxyanthranilic acid **(B)**, xanthurenic acid **(C)**, and 5-hydroxyindolepyruvate **(D)**. Colors indicate the stratum in which correlations were calculated: all (black, full cohort, n = 68), SG (blue, survivors, n = 33), and nonSG (red, non-survivors, n = 35). The x-axis shows the Spearman rho; *, P < 0.05. Proteins shown: **(A)** MYH11, SHROOM3, SEPSECS, NDUFA3, TARS1, and GUSB; **(B)** MMP3, CCDC25, SHROOM3, SH3BGR, BIN1, and AGL; **(C)** B4GALT5, PLSCR3, PMS2P11, XPC, CCL22, and MYOF; **(D)** B4GALT5, MYH11, NDUFA3, SEPSECS, PMS2P11, and GPT.

## 4 Discussion

Severe fever with thrombocytopenia syndrome (SFTS) poses a serious public health threat in East Asia, with substantially higher mortality among critically ill patients than in the general SFTS population. Clinical outcomes in severe SFTS are highly heterogeneous (1–4), reflecting complex host–virus interactions, dysregulated systemic inflammation, and progressive multi-organ dysfunction. In this context, integrated proteomic and metabolomic approaches have emerged as powerful tools for characterizing molecular changes associated with disease progression, identifying potential prognostic biomarkers, and informing the investigation of therapeutic targets in severe viral infections. Accumulating evidence supports the utility of omics strategies for investigating SFTS pathogenesis. Zhao et al. (5) demonstrated that proteomic profiling identifies dysregulated proteins involved in multiple cellular and immune processes in SFTS patients and established a proteasome-centered regulatory axis associated with adverse outcomes. Park et al. (6), by combining targeted proteomics with single-cell transcriptomics, characterized the peripheral blood immune landscape in SFTS and showed that, in fatal cases, SFTSV infection was associated with excessive inflammatory cascades involving proinflammatory cytokines and chemokines, accompanied by aberrant suppression of adaptive immunity. Zhu et al. (7) reported that serum metabolomic profiling of acute-phase SFTS patients linked perturbations in multiple metabolic pathways, particularly sphingolipid metabolism, to poor prognosis. Despite the considerable potential of multi-omics integration in severe viral infections (8–10), few studies have focused specifically on critically ill SFTS patients admitted to the intensive care unit (ICU), and the interplay between metabolic disturbances and proteomic dysregulation in fatal outcomes remains poorly characterized. This study provides the first integrated proteomic–metabolomic analysis of ICU patients with severe SFTS stratified by 28-day outcomes, aiming to characterize the metabolic and proteomic features associated with fatal disease.

Consistent with previous clinical observations (11), baseline characteristics indicated a more severe disease phenotype in non-survivors, including lower Glasgow Coma Scale (GCS) scores, higher SFTSV RNA loads, more pronounced systemic inflammation, multi-organ dysfunction involving the liver, kidney, and myocardium, coagulation disturbances, and reduced CD4^+^ and CD8^+^ T-lymphocyte counts. These findings further support the association of dysregulated host immune responses, systemic inflammation, and multi-organ injury with death in severe SFTS.

The central finding of this study is the marked divergence in metabolite and protein profiles and signaling pathways between survivors and fatal cases. Among the perturbed pathways, tryptophan metabolism showed the strongest enrichment (hsa00380; 10/106 metabolites; adjusted *P* = 0.001), consistent with previous urine metabolomic findings identifying tryptophan metabolism as a major perturbed pathway in SFTS (12). As an essential amino acid, tryptophan participates in diverse physiological processes, including neuronal function, immune regulation, and intestinal homeostasis (13). In mammals, free tryptophan is predominantly catabolized via the kynurenine pathway, which is initiated by the rate-limiting enzymes IDO1 and TDO (14, 15). Our data showed significantly lower L-tryptophan (*P* = 0.0067) and higher levels of kynurenine and its downstream products KA, 3-HAA, XA, and 5-HIP in non-survivors (**Supplementary Information Table S2**). This pattern of lower substrate levels and higher product levels yields a metabolite signature consistent with enhanced kynurenine pathway activity in the setting of infection and critical illness (16–18). In COVID-19 and sepsis, elevated kynurenine-to-tryptophan ratios have been associated with disease severity and mortality (16, 18). Notably, a urine metabolomic study of SFTS likewise identified tryptophan metabolism as the top pathway distinguishing fatal from surviving patients, with elevated 5-HIAA, kynurenine, 5-HTP, and 3-HAA in fatal cases (12), supporting our findings in an independent biospecimen dimension. Importantly, our plasma-based observations suggest, but do not establish, activation of the kynurenine pathway; enzyme activities and metabolic fluxes were not directly measured, a limitation addressed below. Kynurenine pathway metabolites exert broad immunomodulatory effects: kynurenine is an endogenous ligand of the aryl hydrocarbon receptor (AhR) (19); 3-HAA modulates T-cell function and may promote an imbalance between Th17 and regulatory T cells (20, 21); and KA has been positively correlated with inflammatory cytokines and chemokines and negatively correlated with T-cell responses in severe viral infections such as COVID-19 (22). These metabolite-mediated immunomodulatory actions may contribute to immune dysregulation in fatal SFTS. Moreover, the positive correlation of 3-HAA with renal function indicators (BUN and Cys-C) suggests that impaired renal clearance may contribute to metabolite accumulation in fatal cases, a phenomenon also reported in COVID-19, where kynurenine metabolites correlate with renal status and IL-6 levels (17). Altered tryptophan metabolism may thus reflect enhanced IDO/TDO activity together with hepatic and renal dysfunction and the hyperinflammatory, hypercatabolic state of critical illness; these potential mechanisms warrant further investigation.

Beyond tryptophan metabolism, purine catabolism was also associated with fatal SFTS: xanthine and uric acid were significantly higher in non-survivors (**Supplementary Information Table S2**), consistent with the enrichment of nucleotide metabolism. Purine catabolism can generate reactive oxygen species, and uric acid can act as a damage-associated molecular pattern to promote inflammation (23, 24). Accordingly, serum adenosine deaminase (ADA), a key enzyme in purine catabolism that catalyzes the deamination of adenosine to inosine (25), was elevated in fatal SFTS patients and correlated with IL-6, TNF-α, and multi-organ failure (26). Enhanced purine turnover has likewise been observed in COVID-19 non-survivors (27). Together with the elevated purine catabolites in non-survivors, these data support increased purine catabolism in fatal SFTS, although causal roles require functional validation.

Complementing the metabolomic findings, proteomic analysis identified 200 differentially expressed proteins (DEPs) between the two groups. Proteins upregulated in survivors were predominantly enriched in proteasome and neurodegeneration-related pathways, with proteasome enrichment showing the strongest enrichment signal, consistent with Zhao et al. (5). In contrast, proteins upregulated in non-survivors were mainly associated with IL-17 signaling, TNF/NF-κB signaling, chemokine signaling, and cytokine–cytokine receptor interaction pathways, which are involved in inflammation and immune regulation, suggesting more intense inflammatory responses in fatal cases. Notably, four key proteins (SHROOM3, SEPSECS, MYH11, and PIGW) have been reported to have prognostic value (28, 29). These divergent expression profiles suggest that protein homeostasis may be relatively preserved in survivors, whereas fatal cases are characterized by greater activation of inflammatory signaling pathways. Integrative correlation analysis of the four core tryptophan metabolites with 200 DEPs identified a potential metabolite–protein association network in severe SFTS. In the overall cohort, 20 DEPs were significantly correlated with at least one core metabolite (|r| ≥ 0.35, *P* < 0.05), and six associations remained significant after stratification by outcome, including MMP3, SH3BGR, and BIN1, which were negatively correlated with 3-HAA in survivors, and CHL1, PLSCR3, and MYOF, which were correlated with 3-HAA/XA in non-survivors (**Figure 6**; **Supplementary Information Table S5**). These proteins participate in inflammation and matrix remodeling (matrix metalloproteinase 3, MMP3) (30), cytoskeletal organization and intracellular signal transduction (amphiphysin 2, BIN1; SH3 domain-binding glutamate-rich protein, SH3BGR) (31, 32), and membrane dynamics and vesicular trafficking (phospholipid scramblase-3, PLSCR3 (33); myoferlin, MYOF (34)), biological processes associated with systemic inflammatory responses. These findings are correlative; these proteins are not canonical enzymes of tryptophan metabolism, no causal relationship is implied, and their functional links to altered tryptophan metabolic disturbance require experimental validation. Collectively, together with the enrichment of inflammatory proteomic pathways, these results suggest that altered tryptophan metabolism is associated with intracellular protein regulatory networks and the inflammatory microenvironment and may contribute to a molecular signature associated with fatal outcomes in severe SFTS.

ROC analysis showed that the four tryptophan metabolites (KA, 3-HAA, XA, 5-HIP) exhibited good discriminative performance for 28-day mortality, with a combined panel AUC of 0.934 (95% CI: 0.878–0.991), exceeding the AUCs of the conventional clinical indicators evaluated. However, because model development and evaluation were performed in the same cohort with limited sample size, these AUC values should be regarded as exploratory and may be overestimated; the prognostic value of these metabolites requires validation in independent external cohorts. Consistent with these findings, van der Sluijs et al. (35) reported associations between tryptophan metabolites and disease severity and clinical outcomes in viral infection.

This study has several limitations. First, the sample size was relatively small, and the study was conducted at a single center, which may limit generalizability of the findings. Second, both the metabolite–protein correlation and ROC analyses were performed within the same cohort without an independent validation cohort, necessitating external validation of the prognostic value of the findings. Third, incomplete adjustment for comorbidities and medications may have introduced bias. We excluded patients with comorbidities such as malignancy, which may have partly mitigated this bias; moreover, some patients received treatment in general wards or outpatient settings before ICU admission, which may have affected baseline metabolite and protein levels. Fourth, we did not directly measure the activities or metabolic fluxes of key tryptophan-metabolizing enzymes (e.g., IDO1, TDO2, KMO, and KYNU) or perform functional experiments to validate the identified metabolite–protein associations. Future studies should integrate enzyme activity assays, isotope-tracing flux analysis, and *in vitro* and *in vivo* functional experiments to clarify the roles of altered tryptophan and purine metabolism in SFTS pathogenesis and prognosis.

Despite these limitations, this study characterizes the plasma metabolomic and proteomic landscapes of ICU patients with severe SFTS and provides the first evidence linking disturbances in the tryptophan–kynurenine pathway and purine catabolism to 28-day mortality in SFTS, with these metabolic disturbances associated with inflammatory proteomic features. Our findings provide insights into the metabolic and immune processes associated with SFTSV infection and provide an initial foundation for future translational research and therapeutic investigation; these conclusions require further validation in independent cohorts and functional studies.

## Data Availability

The raw proteomic and metabolomic datasets generated and analyzed in this study are publicly available via the Figshare repository at https://figshare.com/s/937eb2c71c5f552e4096.
