# Supplementary material for: Integrated proteomic and metabolomic profiling characterizes tryptophan metabolic disturbance associated with mortality in ICU-admitted SFTS patients
Source: Front Immunol. 2026 Oct 9;17:1898053. doi: 10.3389/fimmu.2026.1898053 (PMC13690963; doi:10.3389/fimmu.2026.1898053)
Supplement: Supplementary file 1 [file DataSheet1.pdf]

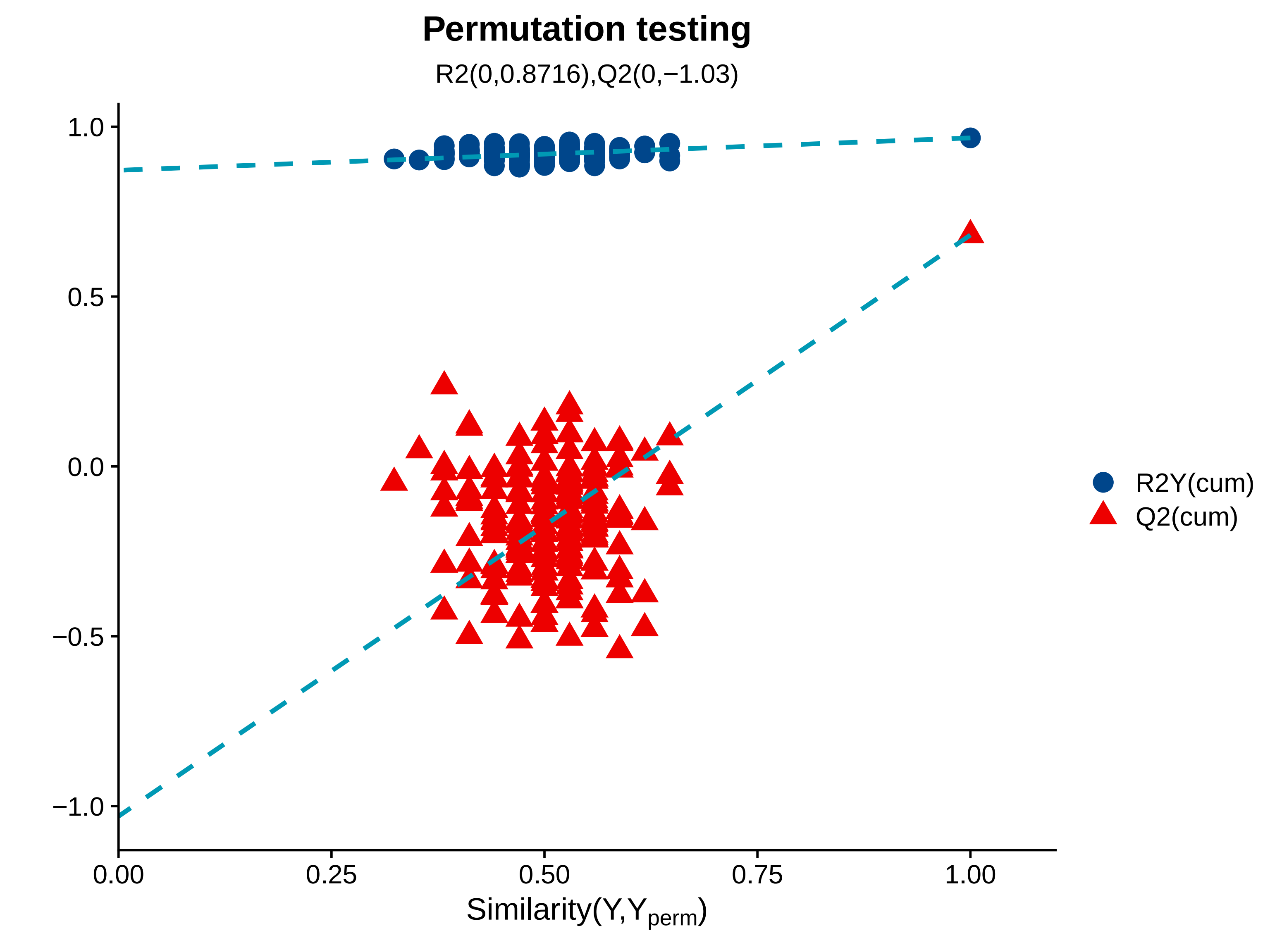
Permutation testing
R2(0,0.8716),Q2(0,−1.03)
1.0
0.5
0.0
−0.5
−1.0
0.00
0.25
0.50
0.75
1.00
Similarity(Y,Yperm)
R2Y(cum)
Q2(cum)
