# Supplementary material for: Integrated proteomic and metabolomic profiling characterizes tryptophan metabolic disturbance associated with mortality in ICU-admitted SFTS patients
Source: Front Immunol. 2026 Oct 9;17:1898053. doi: 10.3389/fimmu.2026.1898053 (PMC13690963; doi:10.3389/fimmu.2026.1898053)

Differentially expressed proteins and tryptophan metabolites (z−score)

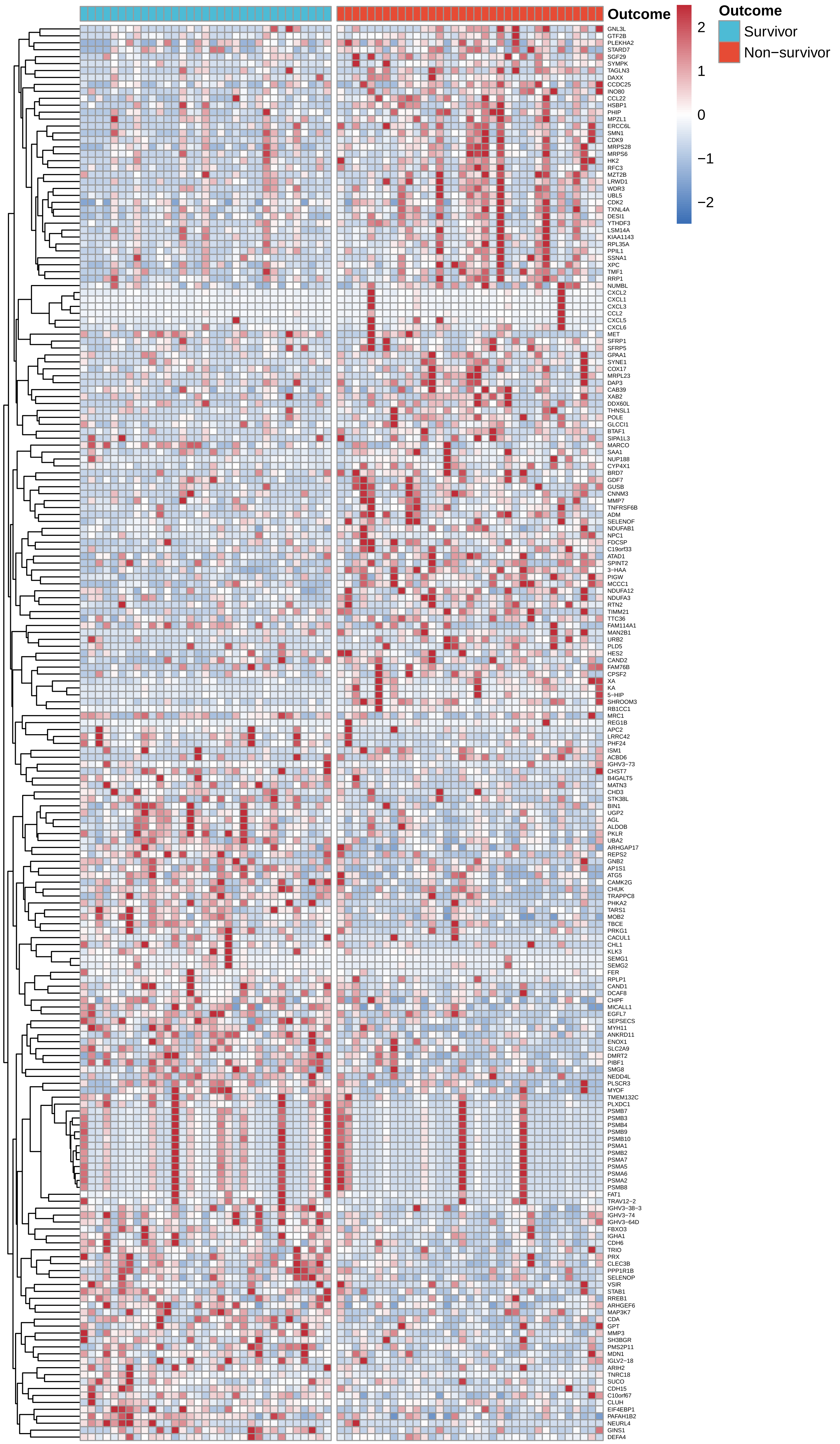

Supplement: Supplementary file 2 [file DataSheet2.pdf]
