# Supplementary material for: Integrated proteomic and metabolomic profiling characterizes tryptophan metabolic disturbance associated with mortality in ICU-admitted SFTS patients
Source: Front Immunol. 2026 Oct 9;17:1898053. doi: 10.3389/fimmu.2026.1898053 (PMC13690963; doi:10.3389/fimmu.2026.1898053)
Supplement: Supplementary file 3 [file Table4.docx]

Chromatographic separation was performed on a SHIMADZU-LC30 ultra-high performance liquid chromatography system equipped with an ACQUITY UPLC® HSS T3 column (2.1×100 mm, 1.8 μm) (Waters, Milford, MA, USA). The injection volume was 16 μL, column temperature 40 ℃, and flow rate 0.3 mL/min. Mobile phase A was 0.1% formic acid in water, and mobile phase B was 0.1% formic acid in acetonitrile. The gradient elution program was as follows: 0.01–1 min, 2% B; 1–5 min, B linearly increased from 2% to 48%; 5–7 min, B linearly increased from 48% to 80%; 7–11 min, B linearly increased from 80% to 100%; 11–13 min, B maintained at 100%; 13–13.01 min, B linearly decreased from 100% to 2%; 13.01–15 min, B maintained at 2%.

Mass spectrometry analysis was performed using a QE Plus mass spectrometer (Thermo Scientific) with electrospray ionization (ESI) in positive and negative modes. Ionization parameters were set as follows: Spray Voltage: 3.8 kv (+) and 3.2 kv (−); Capillary Temperature: 320 ℃ (±); Sheath Gas: 30 (±); Aux Gas: 5 (±); Probe Heater Temp: 350 ℃ (±); S-Lens RF Level: 50. Mass spectrometry acquisition settings were as follows: acquisition time: 15 min; parent ion scan range: 75–1050 m/z; full scan resolution: 70,000 @ m/z 200; AGC target: 3e6; maximum ion time (IT) for full scan: 100 ms. For MS2 analysis, after each full scan, MS2 spectra of the 4 most intense parent ions were triggered. MS2 parameters: resolution: 17,500 @ m/z 200; AGC target: 1e5; maximum IT: 50 ms; activation type: HCD; isolation window: 2 m/z; normalized collision energy (stepped): 20, 30, 40.
