# Supplementary material for: Integrated proteomic and metabolomic profiling characterizes tryptophan metabolic disturbance associated with mortality in ICU-admitted SFTS patients
Source: Front Immunol. 2026 Oct 9;17:1898053. doi: 10.3389/fimmu.2026.1898053 (PMC13690963; doi:10.3389/fimmu.2026.1898053)
Supplement: Supplementary file 4 [file Table5.docx]

The mobile phases were as follows: mobile phase A was 0.1% formic acid in water, and mobile phase B was 0.1% formic acid in 80% acetonitrile. The column was equilibrated with 96% mobile phase A. After sample injection into the Trap Column (PepMap Neo 5 μm C18 300 μm × 5 mm, Thermo Scientific), gradient separation was performed on the analytical column (μPAC Neo High Throughput column, Thermo Scientific). The liquid phase gradient was set as follows: 0–0.1 min, mobile phase B linearly increased from 4% to 6%; 0.1–1.1 min, mobile phase B linearly increased from 6% to 12%; 1.1–4.3 min, mobile phase B linearly increased from 12% to 22.5%; 4.3–6.1 min, mobile phase B linearly increased from 22.5% to 45%; 6.1–8 min, mobile phase B was maintained at 99%.
